# Western Periodicals

**Published:** 1842-06

**Authors:** 


### WESTERN PERIODICALS.

There are now the following medical periodicals in the West:

1. Our own *Western Journal of Medicine and Surgery,* a continuation of the Western Journal of the Medical and Physical Sciences, begun in Cincinnati in the year 1827, and of the Louisville Journal of Medicine and Surgery, begun in 1838—united.

2. The *Western and Southern Medical Recorder,* begun in Lexington, November 1841—not a continuation of the former Transylvania Journal.

3. The *Western Lancet,* begun in Cincinnati, May 1842.

These three journals are published in monthly numbers, the first comprising 80 pages, the second and third each 48, making an aggregate for the year of 2112 pages!

If the West and South should sustain this number of journals, it will speak well for the inquisitiveness and liberality of their physicians. If all who ought to subscribe for and read periodical works should do so, there would be ample support for the whole of these; for the physicians of the valley of the Mississippi amount to thousands. We have heard the multiplication of journals condemned; but who can prevent it, when and where all have an equal right to engage in the enterprise? As well might we attempt to limit the number of newspapers, steamboats, or medical schools. Wherever we have states united into a nation, there are some enterprises that will be multiplied into high competition; which competition, and it only, will apply the corrective. A journal which cannot support itself will not live very long. Like the Irishman's horse, it will eat up its own head; and whatever loses its head, must die. We know not how many carry fodder to our cotemporaries, but the amount contributed to our supply, although not quite sufficient to boast over, or to satiate a bulimious appetite, is enough for good health; and, while we have none to spare, we shall not quarrel with our neighbors, should they even diminish the quantity. If we do not hypocritically hail their appearance in the common field, we shall practise still greater reserve in attempts to drive them from it. We do not feel bound to praise, but we do feel bound not to dispraise their undertakings. These journals are in the interest of the three medical schools in the West; and may be expected, respectively, to advance those interests by every means consistent with *truth* and a *generous* rivalship. *Such* an emulation will do much good and no harm; and we are quite convinced that no other will be cherished. D.

